# Supplementary material for: Maternal constipation is associated with allergic rhinitis in the offspring: A nationwide retrospective cohort study
Source: PLoS One. 2023 Oct 5;18(10):e0292594. doi: 10.1371/journal.pone.0292594 (PMC10553815; doi:10.1371/journal.pone.0292594)
Supplement: S1 Checklist — (DOCX) [file pone.0292594.s001.docx]

STROBE Statement—checklist of items that should be included in reports of observational studies

|  | | Item No. | | Recommendation | Page  No. | | Relevant text from manuscript |
| --- | --- | --- | --- | --- | --- | --- | --- |
| **Title and abstract** | | 1 | | (*a*) Indicate the study’s design with a commonly used term in the title or the abstract | 1 | | Maternal Constipation is Associated with Allergic Rhinitis in  the Offspring: A Nationwide Retrospective Cohort Study |
|  |  |  |  | (*b*) Provide in the abstract an informative and balanced summary of what was done and what was found | 1 | | The results showed that the children whose mothers had constipation had a 1.20-fold risk of AR compared to children of mothers without constipation. Maternal constipation was associated with an increased risk of AR. |
| Introduction | | | | | | |  |
| Background/rationale | | 2 | | Explain the scientific background and rationale for the investigation being reported | 2 | | Alterations in the gut microbiome, a condition known as gut dysbiosis, can lead to dysregulation of immune system maturation, especially in early life. Several risk factors have been identified or proposed for AR, including genetic inheritance or environmental exposures. but the populations at risk of developing into AR is a matter of ongoing research. Among these, microbiota hypothesis is increasingly being discussed in the literature. |
| Objectives | | 3 | | State specific objectives, including any prespecified hypotheses | 2 | | We hypothesize that maternal constipation could influence the risk of AR in offspring and, hence, we analyzed a real-world, population-based retrospective cohort from Taiwan’s National Health Insurance Research Database (NHIRD). |
| Methods | | | | | | |  |
| Study design | | 4 | | Present key elements of study design early in the paper | 3-4 | | See section: Materials and methods:   - Data sources - Study group and outcome - Covariates and matching - Statistical Analysis |
| Setting | | 5 | | Describe the setting, locations, and relevant dates, including periods of recruitment, exposure, follow-up, and data collection | 3 | | This study utilized data obtained from multiple sources, including the National Health Insurance program in Taiwan, birth certificate applications, cause of death data, and the Maternal and Child Health Database. In total, 2,146,530 individuals were enrolled from the birth certificate application database from 2005 and 2015. The study period for data collection was conducted in July 2022. |
| Participants | | 6 | | (*a*) *Cohort study*—Give the eligibility criteria, and the sources and methods of selection of participants. Describe methods of follow-up  *Case-control study*—Give the eligibility criteria, and the sources and methods of case ascertainment and control selection. Give the rationale for the choice of cases and controls  *Cross-sectional study*—Give the eligibility criteria, and the sources and methods of selection of participants | 3 | | Cohort study: 2,146,530 individuals were enrolled from the birth certificate application database from 2005 and 2015.  The Maternal and Child Health Database provides the de-identified number of each mother and child's de-identified number. By linking these databases, we could trace the mother’s comorbidities and medications during pregnancy. |
|  |  |  |  | (*b*) *Cohort study*—For matched studies, give matching criteria and number of exposed and unexposed  *Case-control study*—For matched studies, give matching criteria and the number of controls per case | 3-4 | | Cohort study: The propensity score matching was conducted by birth year, child's sex, birth weight, gestational weeks, delivery mode, mother's comorbidities, and antibiotic use between the two groups. The propensity score was a probability which was estimated through logistic regression, and the binary variable was the maternal constipation and non-maternal constipation group. Matching the propensity score balanced the heterogeneity of the two groups.  Propensity score matching at a 1: 1 ratio was used for the maternal constipation group and the without maternal constipation group, with 102,820 children in each group. |
| Variables | | 7 | | Clearly define all outcomes, exposures, predictors, potential confounders, and effect modifiers. Give diagnostic criteria, if applicable | 4 | | See subsection: Covariate and matching  The baseline characteristics were birth year, child's sex, birth weight… |
| Data sources/ measurement | | 8* | | For each variable of interest, give sources of data and details of methods of assessment (measurement). Describe comparability of assessment methods if there is more than one group | *3-4* | | See section: Materials and methods:   - Data sources - Study group and outcome - Covariates and matching - Statistical Analysis |
| Bias | | 9 | | Describe any efforts to address potential sources of bias | 4 | | The propensity score matching was conducted… |
| Study size | | 10 | | Explain how the study size was arrived at | 4-5 | | 2,146,530 individuals were enrolled from the birth certificate application database from 2005 and 2015. This study utilized data obtained from multiple sources, including the National Health Insurance program in Taiwan, birth certificate applications, cause of death data, and the Maternal and Child Health Database , which is regulated by the Health and Welfare Data Science Center (HWDC) in Taiwan. |
| Quantitative variables | 11 | | Explain how quantitative variables were handled in the analyses. If applicable, describe which groupings were chosen and why | | 4 | See subsection: Statistical Analysis | |
| Statistical methods | 12 | | (*a*) Describe all statistical methods, including those used to control for confounding | | 4 | See section: Materials and methods | |
|  |  |  | (*b*) Describe any methods used to examine subgroups and interactions | | 4 | See subsection: Statistical Analysis | |
|  |  |  | (*c*) Explain how missing data were addressed | | 4 | See section: Materials and methods | |
|  |  |  | (*d*) *Cohort study*—If applicable, explain how loss to follow-up was addressed  *Case-control study*—If applicable, explain how matching of cases and controls was addressed  *Cross-sectional study*—If applicable, describe analytical methods taking account of sampling strategy | | 4 | See section: Materials and methods | |
|  |  |  | (*e*) Describe any sensitivity analyses | | 4 | See subsection: Statistical Analysis | |
| Results | | | | | | | |
| Participants | 13* | | (a) Report numbers of individuals at each stage of study—eg numbers potentially eligible, examined for eligibility, confirmed eligible, included in the study, completing follow-up, and analysed | | 3-5 | See section: Result and Figure 1 | |
|  |  |  | (b) Give reasons for non-participation at each stage | | 3-5 | See section: Result and Figure 1 | |
|  |  |  | (c) Consider use of a flow diagram | | 3 | See Figure 1 | |
| Descriptive data | 14* | | (a) Give characteristics of study participants (eg demographic, clinical, social) and information on exposures and potential confounders | | 16 | See Table 1 | |
|  |  |  | (b) Indicate number of participants with missing data for each variable of interest | | 3 | See Figure 1 | |
|  |  |  | (c) *Cohort study*—Summarise follow-up time (eg, average and total amount) | | 5-6 | See section: Result | |
| Outcome data | 15* | | *Cohort study*—Report numbers of outcome events or summary measures over time | | *5-7,17* | *See Table 2,3,4* | |
|  |  |  | *Case-control study—*Report numbers in each exposure category, or summary measures of exposure | | *-* | *N/A* | |
|  |  |  | *Cross-sectional study—*Report numbers of outcome events or summary measures | | *-* | *N/A* | |
| Main results | 16 | | (*a*) Give unadjusted estimates and, if applicable, confounder-adjusted estimates and their precision (eg, 95% confidence interval). Make clear which confounders were adjusted for and why they were included | | 5-7, 16-17 | See Table 1,2,3,4 | |
|  |  |  | (*b*) Report category boundaries when continuous variables were categorized | | 5-9 | See Table 1,2,3,4,5 | |
|  |  |  | (*c*) If relevant, consider translating estimates of relative risk into absolute risk for a meaningful time period | | - | N/A | |

Continued on next page

| Other analyses | 17 | Report other analyses done—eg analyses of subgroups and interactions, and sensitivity analyses | 6 | See Table 4 |
| --- | --- | --- | --- | --- |
| Discussion | | | | |
| Key results | 18 | Summarise key results with reference to study objectives | 12 | See Section: Conclusion |
| Limitations | 19 | Discuss limitations of the study, taking into account sources of potential bias or imprecision. Discuss both direction and magnitude of any potential bias | 11 | See Section: Discussion |
| Interpretation | 20 | Give a cautious overall interpretation of results considering objectives, limitations, multiplicity of analyses, results from similar studies, and other relevant evidence | 10-11 | See Section: Discussion |
| Generalisability | 21 | Discuss the generalisability (external validity) of the study results | - | N/A |
| Other information | |  | | |
| Funding | 22 | Give the source of funding and the role of the funders for the present study and, if applicable, for the original study on which the present article is based | - | No funding |

*Give information separately for cases and controls in case-control studies and, if applicable, for exposed and unexposed groups in cohort and cross-sectional studies.

**Note:** An Explanation and Elaboration article discusses each checklist item and gives methodological background and published examples of transparent reporting. The STROBE checklist is best used in conjunction with this article (freely available on the Web sites of PLoS Medicine at http://www.plosmedicine.org/, Annals of Internal Medicine at http://www.annals.org/, and Epidemiology at http://www.epidem.com/). Information on the STROBE Initiative is available at www.strobe-statement.org.
