# Supplementary material for: Maternal constipation is associated with allergic rhinitis in the offspring: A nationwide retrospective cohort study
Source: PLoS One. 2023 Oct 5;18(10):e0292594. doi: 10.1371/journal.pone.0292594 (PMC10553815; doi:10.1371/journal.pone.0292594)
Supplement: Attachment — Submitted filename: Comment Letter.docx [file pone.0292594.s002.docx]

Lee et al. demonstrated the relationship between maternal constipation and the development of Allergic Rhinitis (AR) in the offspring. This study contributes to our understanding of the effect of maternal constipation on the risk of AR in offspring. However, there are some concerns regarding the study that need to be addressed.

1. Table 3 illustrates a significantly higher hazard ratio in males. Existing studies generally showed higher constipation prevalence in females (<https://bmcgastroenterol.biomedcentral.com/articles/10.1186/s12876-020-01306-y>). Hence, an in-depth discussion is required to elucidate the increased hazard ratio observed in males.
2. In Table 5, the frequency of laxative usage suggests a potential interpretation: if the frequency is interpreted as constipation severity, a higher risk of allergic rhinitis is seen in mild constipation. This finding warrants further exploration in the discussion section.
3. In the discussion about the potential influence of laxatives, you suggest that a higher dose of laxatives might decrease the risk of developing offspring AR. However, this statement seems speculative and would benefit from supporting evidence or a more in-depth discussion.
4. The section discussing the relationship between mothers with autoimmune diseases and the risk of AR in offspring is a bit unclear. The authors might consider expanding on the rationale behind why mothers with certain autoimmune diseases and constipation show a lower risk of AR in their offspring than mothers without these diseases.
